# Transcriptional Regulation of Mesoderm Genes by MEF2D during Early *Xenopus* Development

**DOI:** 10.1371/journal.pone.0069693

**Published:** 2013-07-19

**Authors:** Alina Kolpakova, Sandra Katz, Aviad Keren, Adi Rojtblat, Eyal Bengal

**Affiliations:** Department of Biochemistry, Rappaport Institute for Research in the Medical Sciences, Faculty of Medicine, Technion-Israel Institute of Technology, Haifa, Israel; University of Colorado, Boulder, United States of America

## Abstract

In *Xenopus*, specification of the three germ layers is one of the earliest developmental decisions occurring prior to gastrulation. The maternally-expressed vegetally-localized transcription factor VegT has a central role in cell autonomous specification of endoderm and in the generation of mesoderm-inducing signals. Yet, marginally-expressed transcription factors that cooperate with mesoderm-inducing signals are less investigated. Here we report that the transcription factors MEF2A and MEF2D are expressed in the animal hemisphere before mid-blastula transition. At the initiation of zygotic transcription, expression of MEF2D expands into the marginal region that gives rise to mesoderm. Knockdown of MEF2D delayed gastrulation movements, prevented embryo elongation at the subsequent tailbud stage and caused severe defects in axial tissues. At the molecular level, MEF2D knockdown reduced the expression of genes involved in mesoderm formation and patterning. We also report that MEF2D functions with FGF signaling in a positive feedback loop; each augments the expression of the other in the marginal region and both are necessary for mesodermal gene expression. One target of MEF2D is the *Nodal-related 1* gene (*Xnr1*) that mediates some of MEF2D mesodermal activities. Chromatin immunoprecipitation analysis revealed that MEF2D associates with transcriptional regulatory sequences of the *Xnr1* gene. Several MEF2 binding sites within the proximal promoter region of *Xnr1* were identified by their *in vitro* association with MEF2D protein. The same promoter region was necessary but not sufficient to mediate MEF2D activity in a reporter gene assay. In sum, our results indicate that the MEF2D protein is a key transcription factor in the marginal zone acting in a positive feedback loop with FGF signaling that promotes mesoderm specification at late blastula stages.

## Introduction

Specification of the three primary germ layers in *Xenopus* embryos is one of the earliest developmental events occurring before the end of the blastula stage [1]. In this process, vegetal, marginal and animal cells are specified to form endoderm, mesoderm and ectoderm, respectively. Since zygotic transcription is initiated only at the 12^th^ cell cycle (mid blastula transition; MBT), maternal mRNA inherited from the oocyte plays a significant role in early specification and patterning [2]. Maternal transcripts encode transcription factors and signaling molecules and are often localized to specific areas of the early embryo such as the vegetal pole or the animal pole [3]. For example, the T box transcription factor, VegT, is localized in the vegetal hemisphere and plays a central role in endoderm and mesoderm specification [4]. VegT and the dorsally-expressed β-catenin induce the transcription of nodal related genes, *Xnr5* and *Xnr6*, at the 256 cell stage [5]. At MBT, these nodal-related genes induce the non-autonomous expression of *Xnrs* 1, 2 and 4 that is essential for mesoderm induction [6], [7]. As a result, *Xnr* genes are expressed at higher levels on the dorsal side of the embryo (Nieuwkoop center), creating a dorsal-ventral gradient in late blastula stages. Nodal signaling induces mesoderm identity and patterning in the overlying marginal zone [8], [9]. Fibroblast growth factor (FGF) signaling that is initiated at late blastula in the marginal zone, induces the expression of the *brachyury* gene (XBra) that encodes a key transcription factor in mesoderm specification [10], [11], [12], [13], [14], [15]. Yet, the transcription programs responsible for Nodal and FGF signaling in the specification and patterning of mesoderm are still largely unknown.

The MEF2 (Myocyte Enhancer Factor 2) family of transcription factors is involved in many aspects of cell differentiation and organogenesis. There are four members of MEF2 in vertebrates: MEF2A, B, C and D. Their conserved DNA binding and dimerization motifs mediate binding of homo- or hetero-dimers to a consensus A/T rich DNA sequence [16].

Three isoforms of MEF2 have been characterized in *Xenopus*: MEF2A, MEF2D and MEF2C. Early studies demonstrated the involvement of MEF2A and MEF2D in somitic skeletal muscle development and cardiac gene expression [17], [18]. An additional role of MEF2A in the formation of anterior neural structure downstream to the Nemo like kinase (NLK) was reported recently [19]. The effects of MEF2A were attributed to its expression during neural development. Unlike MEF2A and MEF2D, MEF2C does not participate in muscle development, but in the development of tendons where it cooperates with Xscleraxis [20]. Interestingly, the expression of MEF2A and MEF2D is detected before MBT, unlike the expression of MEF2C that is initiated at zygotic stage 13 [17], [20]. Early expression of MEF2A and MEF2D proteins may indicate their early function in germ layer specification and patterning.

In the present study we investigated early expression patterns of MEF2A and MEF2D and their possible involvement in germ layer specification. Our results indicate that the two genes are maternally expressed and localized to animal cells. Following MBT, zygotic expression of MEF2D expands into the marginal region. The role of MEF2D at the blastula stage was studied by its antisense morpholino-mediated knockdown. It was found that absence of MEF2D prevented normal development of axial organs of mesoderm and ectoderm origin. We also show that the expression of MEF2D in the marginal zone was necessary for the response of this tissue to vegetal signals and the induction of mesoderm gene expression. We report that MEF2D is involved in the expression of many mesodermal genes including that of signaling molecules of the BMP, Nodal and FGF pathways. MEF2D and FGF signaling maintained each other’s expression, constituting a positive feedback loop. Lastly, we show that MEF2D associates directly with regulatory sequences of one target gene of the Nodal family, *Xnr1*. Thus, our study uncovers the involvement of MEF2D in the establishment of early mesoderm.

## Materials and Methods

### Ethics Statement

All *Xenopus* laevis experiments in this study have been conducted under protocol # **IL-** 149-12-2010 that is valid until December 2014 and approved by the Technion Committee for Care and Use of Laboratory Animals. The Technion holds a valid assurance (#A5026-01) of the US Department of Health and Human Services for humane care and use of laboratory animals.

### Ovulation and Fertilization

Female *Xenopus laevis* frogs were injected with human Chorionic Gonadotropin (hCG, 1000 units per frog) 24 h prior to fertilization. Following fertilization in vitro, eggs were maintained in Modified Ringers (1xMR) salt solution. Fertilized eggs were de-jellied with 2% L-Cysteine solution, washed in 1/3xMR, and injected in 3% Ficoll with different concentrations of capped sense in vitro transcribed RNA during the single cell stage. Embryos were staged according to [21]. Animal Cap (AC) and Vegetal Pole (VP) explants were removed at stage 9 using watchmakers’ forceps. Dorsal Marginal Zone (DMZ) and Ventral Marginal Zone (VMZ) explants were removed at stage 10.5 with an eyebrow knife. Explants were cultured in 1XLCMR solution with gentamycin (50 mg/ml) until sibling embryos reached the desired stage.

### Recombinant Explants

AC explants excised at stage 9 from naïve and XMEF2D AMO-microinjected embryos were conjugated with naïve vegetal pole explants (excised at the same time) in 1xLCMR solution with gentamycine, at 13°c for 3 hrs. AC and vegetal explants were then separated; AC explants were cultured until sibling embryos reached stage 12 and subjected to RT-PCR analysis.

### Microinjections

Capped sense in vitro transcribed RNA encodingMef2-VP16 (1 ng), MEF2D-Flag (1 ng),MEF2D-eng (1 ng) and two Antisense Morpholino Oligonucleotides (AMOs) from Genetools, XMEF2D (30, 20 or 10 ng) (5′-GGATCTTTTTTCTGCCCATGATTCC-3′) and misXMEF2D (30, 20 or 10 ng) (5′- GGAaCaTTTTTgTGCCgATcATTgC-3′), were microinjected either marginally, animally or vegetally into single-cell embryos in 3% Ficoll solution. Reporter gene plasmid (3xMef2-Luc) was injected at a concentration of 30 pg per embryo. Following microinjection embryos were cultured overnight in 3% Ficoll solution, washed in 1/3MR solution and cultured until sibling untreated embryos reached the desired stage. MEF2C-VP16: was described [22]. The coding region was inserted into the pCS2 plasmid. MEF2D-En^R^: Human MEF2D was PCR isolated and inserted into BamH1-EcoR1 sites of the ENG-N plasmid which is a pCS2 based vector encoding for the engrailed repressor domain. MEF2D-Flag: Reverse transcriptase PCR was used to amplify human and Xenopus MEF2D from mRNA using specific primers. The reverse primer skipped the MEF2D termination codon and encoded for an in frame flag peptide. Fragments were cloned into pCS2 plasmid.

### RT-PCR

Analysis was performed as described [23], except that random hexamers (100 ng per reaction) were used for reverse transcription. Primers are described in http://www.xenbase.org.

### Real Time RT-PCR

Total RNA extraction and cDNA synthesis was performed as described above. Quantitative real-time PCR was carried out using Stratagene Mx3000P device and SYBR Premix Ex Taq II reaction mix (TaKaRa). The PCR conditions were as follows: an initial 2-min induction step at 95°C, followed by 40 cycles of amplification: 20-sec at 95°C, 30-sec at 55°C, and 30-sec at 72°C. The run is finished by a melting curve from 95°C to 55°C to ensure PCR product purity. Two negative controls were used to verify the authenticity of the results: reverse transcriptase negative control from the cDNA synthesis step and water-only control. In all cases those negative controls failed to produce specific products. *Ornithine decarboxylase* (*ODC*) was used as a loading control and all values were normalized to its levels. Each experiment was repeated a minimum of three times in independent experiments to verify reproducibility of results. Primer sequences used to amplify cDNA are presented in **Table 1**.

**Table 1:** Pairs of primers used in reverse transcriptase qPCR analysis.

*Bmp4*	F	5′-	GCATGTACGGATAAGTCGATC -3′
	R	5′-	GATCTCAGACTCAACGGCAC -3′
*Chrd*	F	5′-	AACTGCCAGGACTGGATGGT -3′
	R	5′-	GGCAGGATTTAGAGTTGCTTC -3′
*Fgf4*	F	5′-	AATGGCATGCACAGTGAAAA -3′
	R	5′-	TCCATACAGCTTCCCCTTTG -3′
*Fgf8*	F	5′-	TGGTGACCGACCAACTAAGC -3′
	R	5′-	ACGATTAACTTGGCGTGTGG -3′
*Gsc*	F	5′-	TTCACCGATGAACAACTGGA -3′
	R	5′-	TTCCACTTTTGGGCATTTTC -3′
*Mef2A*	F	5′-	CCACAGCAAACCTTTCCAAT -3′
	R	5′-	GATGCACTGGCAGCTAATGA -3′
*Mef2D*	F	5′-	AGGAGGAGTTTCACAAGCGA -3′
	R	5′-	GAGTGACACCGGGATGAGTT -3′
*Mixer*	F	5′-	CACCAGCCCAGCACTTAACC -3′
	R	5′-	ATCCAGCTTGTTCTGGCTGT -3′
*Myf5*	F	5′-	CTATTCAGAATGGAGATGGT -3′
	R	5′-	GTCTTGGAGACTCTCAATA -3′
*Noggin*	F	5′-	AGTTGCAGATGTGGCTC -3′
	R	5′-	AGTCCAAGAATCTCAGC -3′
*ODC*	F	5′-	GCCATTGTGAAGACTCTCTCCATTC -3′
	R	5′-	TTCGGGTGATTCCTTGCCAC -3′
*Szl*	F	5′-	GTCTTCCTGCTCCTCTGC -3′
	R	5′-	AACAGGGAGCACAGGAAG -3′
*VegT*	F	5′-	CAAGTAAATGTGAGAAACCGTG -3′
	R	5′-	CAAATACACACACATTTCCCGA -3′
*Vent1*	F	5′-	CCAAGGAGAAAGGATGGACA -3′
	R	5′-	CCATTGTGGTCAGTGTCCTG -3′
*Wnt8*	F	5′-	TATCTGGAAGTTGCAGCATACA -3′
	R	5′-	GCAGGCACTCTCGTCCCTCTGT -3′
*Xbra*	F	5′-	TTCTGAAGGTGAGCATGTCG -3′
	R	5′-	GTTTGACTTTGCTAAAAGAGACAGG -3′
*Xnr1*	F	5′-	TGTCGAAAATGGGAAACCTC -3′
	R	5′-	GTGGTGCCTCAAAACAACCT -3′

### Western Blot Analysis

Protein extracts were prepared as described [24]. Equal amounts of extracted proteins (40 µg) were loaded, separated by SDS-polyacrylamide gel electrophoresis and transferred to nitrocellulose filters (1 hr, 200 mA). Membranes were incubated with the following antibodies (o/n, 4°c): anti-Mef2 (C21 Santa Cruz); anti-Tubulin (Sigma), and anti-Flag (M2: Sigma). Bound antibodies were detected with HRP-conjugated secondary antibodies (goat anti-rabbit or goat anti-mouse; Thermo Scientific) diluted 1∶2500 in blocking solution, and visualized using the enhanced chemiluminescence kit (Pierce).

### Histology

Slides were deparaffinized and hydrated with decreasing alcohol concentrations. The hematoxylin-eosin method was used to stain the tissue. Slides were analyzed by optical microscopy.

### In situ Hybridization (ISH) and Immunohistochemistry

ISH was carried out with digoxigenin-labeled probes [24], [25], [26]. Embryos were cultured to different stages and subsequently fixed for *in situ* hybridization. *Mef2a, Mef2d, goosecoid, chordin, myod*, *Xnr1* and *brachyury* antisense probes were used. For immunohistochemistry on sections, slides were deparaffinized and hydrated with a decreasing alcohol gradient. Then sections were blocked with goat serum for 1 hour followed by 14 h incubation at 40°C with anti-MEF2 antibody (Santa Cruz C21, 1∶100). This was followed by incubation with an appropriate biotinylated secondary antibody, streptavidin-peroxidase conjugate, and S-(2-aminoethyl)-Lcysteine (AEC) as a substrate (Histostain-SP kit; Zymed Laboratory, San Francisco, CA, USA); counterstaining was done with hematoxylin.

### Electrophoretic Mobility Shift Assay (EMSA)

Embryos were injected with mRNA encoding MEF2D- Flag (1 ng per embryo). Fifty stage 9 embryos were lysed for 15 min at 4°C using lysis buffer (20 mMTrisbase pH7.6, 10 mM KCl, 5 mM MgCl_2,_ 300 mM Sucrose, 10 mM Glycerol Phosphate,0.2 mM EGTA, 0.5% NP40, 0.5 mM DTT). Lysates were centrifuged (5 min, 2000 rpm, 4°c). The supernatant was then collected and re-centrifuged (20 min, 14,000 rpm, 4°C). The resulting supernatant served as the protein extract. The double-stranded DNA probe containing a MEF2 binding site from the MCK enhancer region was used as a positive control for MEF2 binding. The sequences of the sense strand of DNA probes were, MCK: 5′-AGCTCGCTCTAAAAATAACCCTGGATCC-3′. DE: 5′ CTGTATATTTATATCTATTTATTATAATGC 3′ IE2∶5′ GTAGAGCTTTATATATAAAACTTATAC 3′ IE1∶5′ CACCAGATTATTATTATTTTAGAC 3′ PE: 5′ GCTCACTCCTATATAAAGTCAGGG 3′. Double-stranded probes were labeled by a “filling in” Klenow reaction with [^32^P-α] dCTP. Labeled probes were then separated with Probe Quant G-50 Micro-Clolumns (GE Healthcare). Protein extracts (20 µg) in binding buffer (10% glycerol, 2 mM spermidine, 0.1 mg/ml bovine serum albumin, 2 mM MgCl_2_, 0.02% Nonidet P-40, 0.1 mM EDTA, 50 mM KCl, 10 mM HEPES, pH 7.9) were incubated with unlabeled competitor DNA (500 ng of poly(dI-dC)-poly(dI-dC)) or MEF2 antibody (2 µl, Anti-MEF2, Santa Cruz C21) on ice for [30 min] prior to the addition of the radioactive probe. The labeled probe (50,000 cpm, ∼1 ng) was then added and the mixture was incubated for 30 min at room temperature. DNA-protein complexes were then resolved on a 4% native polyacrylamide gel (0.25× TBE).

### Chromatin Immunoprecipitation

Chromatin immunoprecipitation assays were performed as described [27]. Embryos were injected at the one cell stage with mRNA encoding MEF2D-Flag (1.5 ng per embryo). A second group of control embryos were not injected. Embryos were collected at stage 10 (50 embryos/sample) and processed according to the protocol. Antibodies used to immunoprecipitate chromatin were polyclonal anti-Flag (“specific”) and Rabbit pre-immune serum (“non-specific”). Sequences of primers used to amplify the immunoprecipitated fragmented DNA are presented in **Table 2**. Data was analyzed by the ΔΔC(t) (or Livak) method as described [27].

**Table 2:** Pairs of primers used in Chromatin IP analysis.

*Tubulin intron*	F	5′-	GGGATTTCGGCCTTTTTCAG -3′
	R	5′-	ATTTACCTCCCTGCCGAACC -3′
*Xnr1 (-1904)*	F	5′-	CGCAACGATAAAATGCAATG -3′
	R	5′-	TTTTCGGCAAAGTGAAAAGG -3′
*Xnr1 (-1388)*	F	5′-	TCTTGCACCATATGCAAACC -3′
	R	5′-	TGCCTGAAACAGATTTGCTG -3′
*Xnr1 (-962)*	F	5′-	AGCTTCTCAAAGGGGTTGGT -3′
	R	5′-	CCCCTTTGGACTCCATCTTT -3′
*Xnr1 intron 1*	F	5′-	TTGTTGCACCTTAAATTGTTGG -3′
	R	5′-	TGTTGGAGATGCCATGATAAA -3′

• Numbering is relative to translation initiation codon of Xnr1.

### Luciferase Assay

A 696 bp fragment including proximal promoter sequences of *Xnr1* was PCR-amplified from genomic *Xenopus* DNA and was inserted into the pGL2-basic vector (Promega). The correct sequence was verified by sequencing of the plasmid. This reporter gene or x3 Mef2-Luc were either injected to embryos (20 pg DNA) or transfected to 293T HEK cells (2 µg DNA). Several MEF2-expression plasmids were co-transfected with each of the reporter genes. Extract preparation and luciferase assay were performed using the Luciferase Assay System (Promega). Proteins were extracted from transfected cells (6 cm plate in 500 µl buffer) or embryos injected with luciferase reporter gene using the Reporter Lysis Buffer (Promega, 10 embryos in 200 µl buffer). Luciferase activity was measured by integrating total light emission over a period of 30 sec using a Berthold luminometer. Luciferase activity was normalized to total protein concentration.

### Statistical Analysis

The graphs in the figures are presented as means ± S.E. Statistical significance was analyzed using the Student’s t-test. In the figures, * indicates p<0.05, ** p<0.01, *** p<0.001.

## Results

### Maternal MEF2 is Localized at the Animal Hemisphere of the Embryo

**Figure 1:**
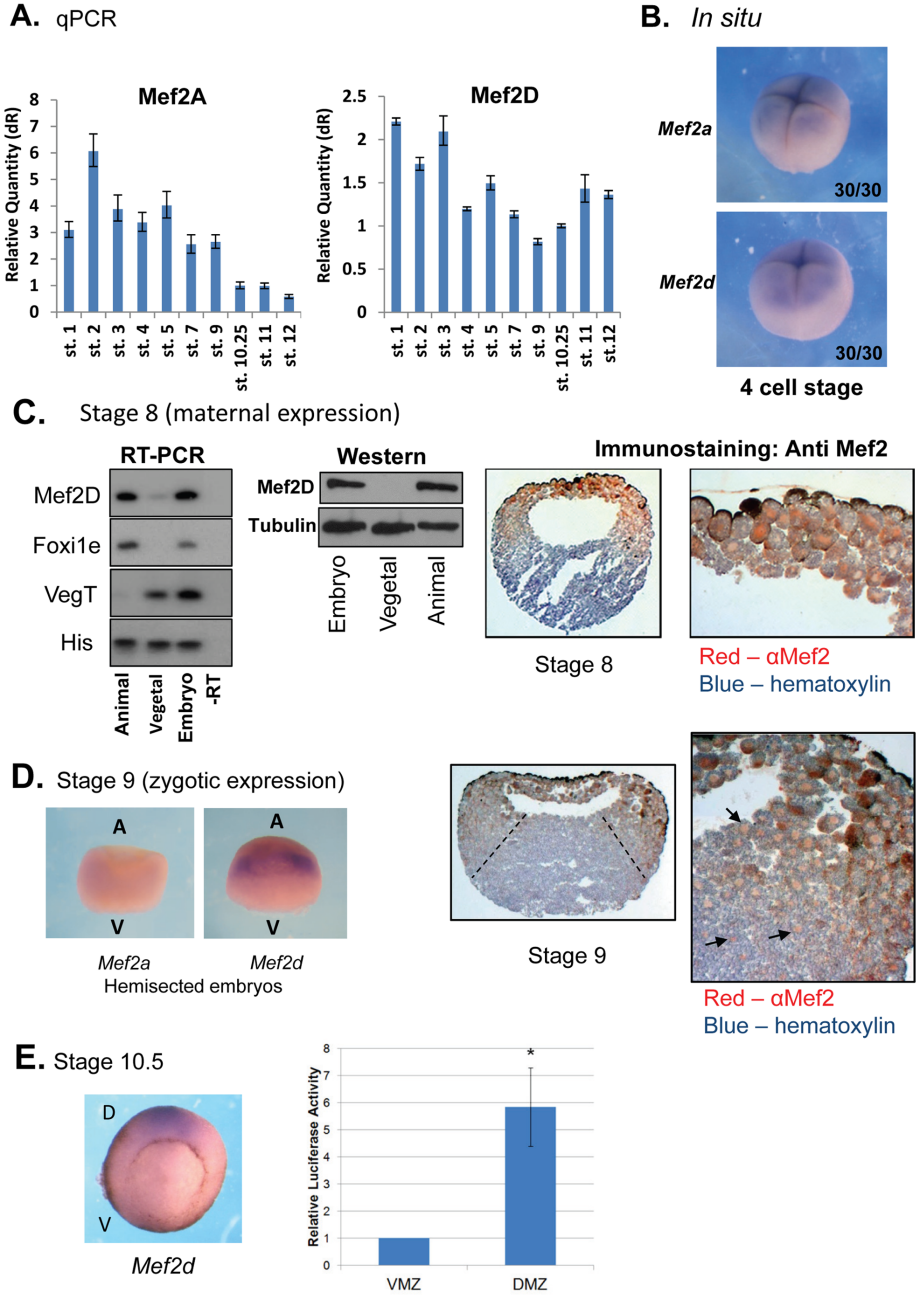
Expression pattern of maternal and zygotic MEF2. (A) Quantitative PCR analysis of MEF2A and MEF2D transcripts from fertilization to gastrula stages. Experiment was performed two independent times. (B) 4 cell embryos were analyzed by ISH using probes *to mef2a* and *mef2d*. (C) Left panels: Stage 8 embryos were dissected to animal and vegetal halves. RNA and proteins were extracted. RNA was analyzed by RT-PCR reaction and proteins by Western analysis. Right panel: Immunohistochemistry of stage 8 embryos. Animal-vegetal sections were prepared. Sections were reacted with anti-MEF2 antibodies (orange) and counterstained with hematoxylin. The right panel is an enlargement of a segment of the left panel. (D) Left panel: Hemisected stage 9 embryos were analyzed by ISH using probes *to mef2a and mef2d*. Right panel: Sections of stage 9 embryos were reacted with anti-MEF2 antibodies (orange) and counterstained with hematoxylin. The right end panel is an enlargement of a segment of the left panel. Arrows point at stained nuclei. (E) Left panel: Stage 10.5 embryos were analyzed by *in situ* hybridization using a probe *to mef2d*. Right panel: x3 Mef2-Luc reporter (30 pg) was injected to one cell embryo (20 embryos). At early gastrula stage (10.25), DMZ and VMZ explants were isolated and luciferase was measured. Luciferase activity was normalized to the total protein levels. Data are presented as means ± SE of three independent experiments.

**Figure 2:**
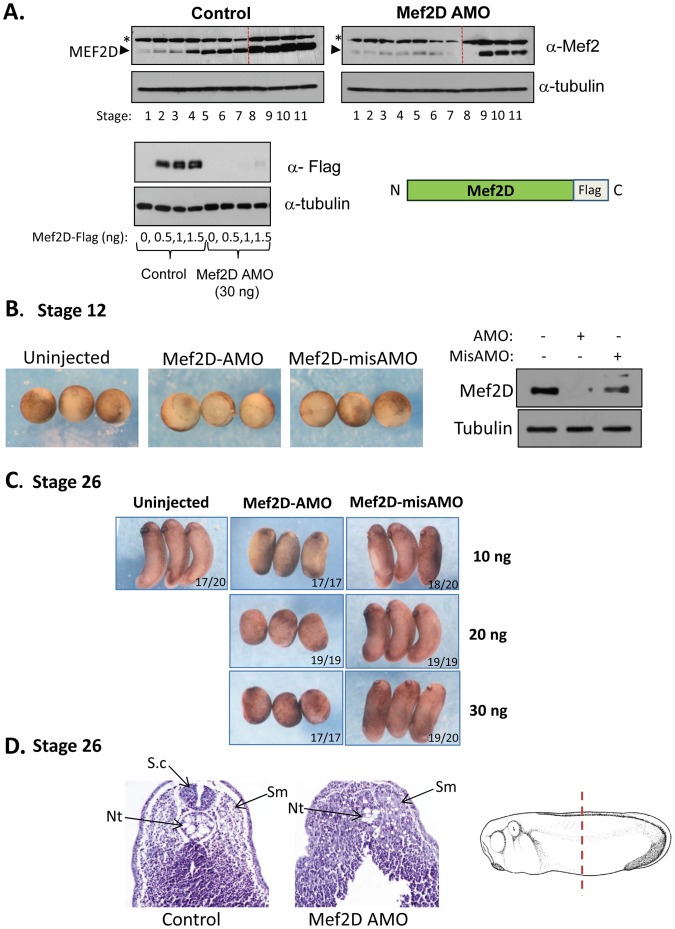
Axial abnormalities in MEF2D-knockdown embryos. (A) Upper panel: Western blot analysis of MEF2D protein extracted from control embryos and MEF2D AMO-injected embryos at different stages from fertilization to late gastrula stages. Lower panel: Embryos were injected with different concentrations of MEF2D-Flag mRNA, without or with AMO to MEF2D. MEF2D-Flag protein was detected by Western blot analysis using anti Flag antibodies (M2, Sigma). (B) Control uninjected embryos, MEF2D AMO-injected embryos and mismatch-AMO injected embryos. Left panel: Stage 12 embryos; MEF2D AMO delays blastopore closure. Right panel; Western blot of endogenous MEF2D from uninjected, MEF2D AMO and mismatch AMO-injected embryos. (C) Stage 26 embryos that were injected with different amounts of MEF2D AMO and mismatch AMO. (D) Transverse sections in the trunk region of stage 26 control uninjected embryo (left panel) and MEF2D AMO-injected embryo (right panel). The scheme at the right shows the position where sections were performed. Abbreviations: Sm-Somite; Nt-Notochord; S.c-Spinal cord.

The expression levels of the *mef2a* and *mef2d* gene transcripts from fertilization until gastrula stages were analyzed by quantitative PCR (qPCR). In agreement with previous studies [17], [20], pre-MBT maternal transcripts of both isoforms were detected (Figure 1A). These transcripts were localized animally at the 4 cell stage (Figure 1B). While *mef2a* expression levels declined after MBT, zygotic *mef2d* transcripts progressively accumulated due to the onset of zygotic transcription (Figure 1A). *Mef2c* was not detected by qPCR at these stages (data not shown) [20]. Localization of the *mef2d* mRNA and protein was analyzed in embryos at stages 8 and 9. Reverse transcription-PCR and Western analyses indicated that MEF2D was confined to the animal hemisphere at stage 8 (Figure 1C, left panels). Likewise, sections that were immunostained with anti MEF2 antibody also indicated animal expression of MEF2D (Figure 1C, right panel). This antibody detected a single band of the MEF2D expected size that was diminished in embryos that were injected with specific antisense morpholino to *mef2d* (see Figure 2A). In addition, the same anti MEF2 antibody specifically and robustly stained cell nuclei of early neurula stage paraxial mesoderm as was expected for MEF2D (Figure S1). At stage 9, both *in situ* hybridization (ISH) and immunostaining analyses indicated that the expression of zygotic MEF2D was expanded into the marginal region (Figure 1D). At stage 10.5, *mef2d* transcript was localized to the dorsal marginal zone (DMZ) (Figure 1E, left panel). At that stage, the expression of injected Mef2-reporter gene (x3 Mef2 Luc) indicated that MEF2 activity was enriched in the DMZ relative to the ventral marginal zone (VMZ) (Figure 1E, right panel). *In situ* hybridization at later stages revealed zygotic expression of *mef2d* in the paraxial mesoderm, while expression of *zygotic mef2a* was detected first at stage 15 (Figure S2). In conclusion, expression of MEF2D before MBT is confined to the animal half of embryos whereas at late blastula it expands to the marginal zone. At early gastrula stage MEF2D expression is reduced to the DMZ and in the latter neurula stages it takes place in the paraxial mesoderm.

### Morpholino-mediated Knockdown of MEF2D Interferes with Axial Development

To study the involvement of MEF2D protein in early specification and patterning, antisense morpholino (AMO) to MEF2D was injected to single-cell embryos. Injected mismatched MEF2D AMO served as controls. Injection of MEF2D AMO significantly reduced the expression of endogenous MEF2D protein at different developmental stages before and following MBT (Figure 2A). In addition, MEF2D AMO prevented the ectopic expression of *Xenopus* MEF2D-Flag protein (Figure 2A). Injection of MEF2D AMO delayed blastopore closure relative to uninjected or mismatch AMO-injected embryos (Figure 2B). Mismatch MEF2D AMO mildly-reduced MEF2D protein levels (Figure 2B, right panel). This result might explain the subtle effects of injected mismatch AMO relative to uninjected embryos that were observed in the rest of the study. Increasing amounts of injected MEF2D AMO induced significant shortening of the body axis whereas the mismatch-AMO displayed a minimal effect on embryo elongation at stage 26 (Figure 2C). Histological sections of stage 26 embryos revealed that axial structures were significantly affected in MEF2D-depleted embryos (Figure 2D). The spinal cord was absent while the structures of the notochord and somites were greatly reduced. In conclusion, these results indicated that MEF2D is involved in gastrulation movements and the formation of axial organs of mesoderm and ectoderm origin.

### MEF2D is an Inducer of Mesoderm and the Spemann Organizer

**Figure 3:**
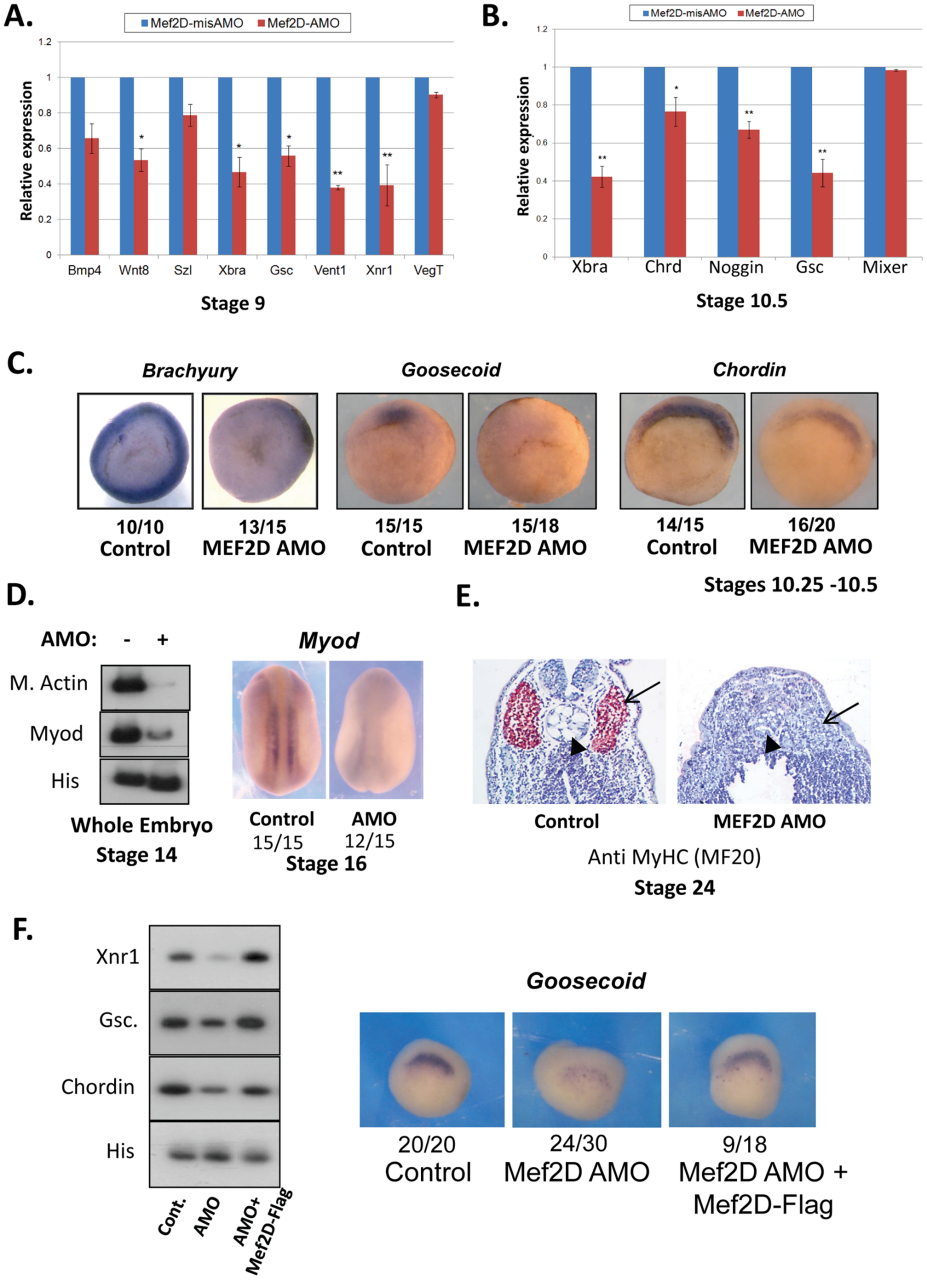
MEF2D regulates the expression of mesoderm genes. (A) qPCR analysis of embryos injected with MEF2D AMO or mismatch AMO. 5 embryos on each group were injected and RNA was extracted at stage 9. qPCR was performed as described in “materials and methods”. Expression levels of each gene were arbitrarily set to a value of 1 in the mismatch AMO injected embryos. The values for each gene were standardized accordingly. Data are presented as means ± SE of two independent experiments with duplicates(B) qPCR was performed on stage 10.5 embryos treated as is described in A. (C) Stage 10.5 embryos were analyzed by ISH using antisense probes to *brachyury*, *goosecoid* and *chordin* (left to right). (D) Control and MEF2D AMO-injected embryos were analyzed at stage 14 by RT-PCR (left) and at stage 16 by ISH with antisense probe to *myod*. (E) Transversal sections of stage 24 control and MEF2D AMO-injected embryos analyzed by immunohistochemistry. The antibody used was anti Myosin heavy chain (MF20) and samples were counterstained with hematoxylin. (F) Co-injection of MEF2D-Flag mRNA with MEF2D-AMO restores the expression of mesoderm and organizer genes. MEF2D AMO was injected alone or together with Mef2D-Flag mRNA into one cell embryos. Embryos were analyzed by RT-PCR (left) and by ISH with antisense probe to *goosecoid* (right) at stage 10.25.

Expression of MEF2D in the marginal zone starts at the late blastula stage, and the loss of axial mesodermal structures including the somites and notochord in MEF2-depleted embryos suggested that mesoderm might be affected by MEF2D. To investigate this possibility, MEF2D AMO and control mismatch AMO were injected to one cell embryos and expression patterns of several mesodermal genes were analyzed by qPCR (Figure 3A). At late blastula stage, the expression of early mesodermal genes was significantly reduced whereas the expression of the vegetal gene, *vegt* was not affected in MEF2D AMO-injected embryos (Figure 3A). At gastrulae, expression of Spemann organizer genes, *goosecoid, chordin* and *noggin* was diminished in MEF2D-depleted embryos while that of the vegetal gene, *mixer* was not affected (Figure 3B). At this stage, the normal ring-like expression of *brachyury (Xbra)* around the blastopore was barely detected and organizer expression of *goosecoid* and *chordin* was significantly reduced in the MEF2D AMO-injected embryos (Figure 3C). At the neurula stage, the paraxial expression of *myod* and *muscle actin* was substantially decreased in MEF2D AMO-injected embryos relative to control embryos (Figure 3D). At the tailbud stage, the expression of myosin heavy chain was completely absent in MEF2D AMO-injected embryos, yet the somite and notochord structures are observable but smaller than in control embryos (Figure 3E). It therefore appeared that MEF2D was required for the early expression of mesoderm inducers such as *Xnr1* and *Xbra*, as well as for the subsequent expression of organizer and paraxial mesoderm genes such as *chordin, noggin, myod* and *muscle actin.* The specificity of MEF2D knockdown was investigated by injection *of mef2d* mRNA containing three mismatches along the AMO-targeted sequence. Co-injection of this transcript rescued the expression of mesoderm genes in MEF2D-knockdown embryos (Figure 3F). In a second approach to block MEF2 activity, a chimera protein composed of MEF2D and the Engrailed repressor domain of *drosophila* (MEF2D-En^R^) was expressed in embryos. Injection of *mef2d-en* ^R^ mRNA repressed the promoter activity of a MEF2-dependent reporter gene (x3 Mef2-Luc) suggesting that MEF2D-En^R^ chimera functioned as a dominant negative of MEF2 activity (Figure S3). It also decreased transcript levels of the mesodermal genes, *Xbra*, *goosecoid, noggin* and *chordin* in a concentration-dependent manner (Figure S3).

### Ectopic MEF2 Activity Induces the Expression of Mesoderm Genes

**Figure 4:**
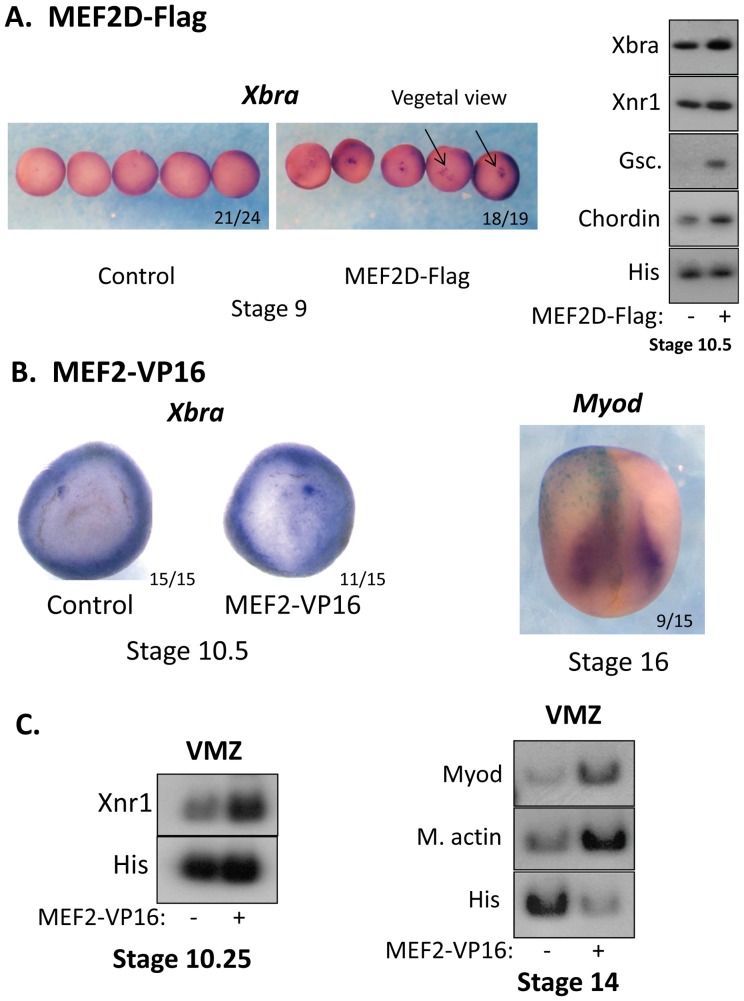
Gain of MEF2D function induces the expression of paraxial mesoderm genes. (A) ISH of stage 9 vegetally-injected embryos (Mef2D-Flag mRNA) using antisense *Brachyury* probe (left). Arrows point at punctate staining. MEF2D-Flag mRNA was injected marginally to one cell embryos. RNA was extracted from whole embryos (n = 18) at stages 10.5 and the expression levels of the indicated genes was analyzed by semi quantitative RT-PCR (right). (B) mRNA encoding MEF2-VP16 chimera was injected to one cell embryos. Left panel: Stage 10.5 embryos were analyzed by ISH using antisense probe to *brachyury*. Right panel: Two blastomere embryos were injected unilaterally with MEF2-VP16 and βGAL mRNA and grown to stage 16. ISH with a probe to *myod* was performed. The injected side was identified by βGAL staining. (C) MEF2-VP16 mRNA was injected marginally to one cell embryos. RNA was extracted from VMZ explants (n = 18) at stages 10.25 (left) and 14 (right) and analyzed by semi quantitative RT-PCR.

**Figure 8:**
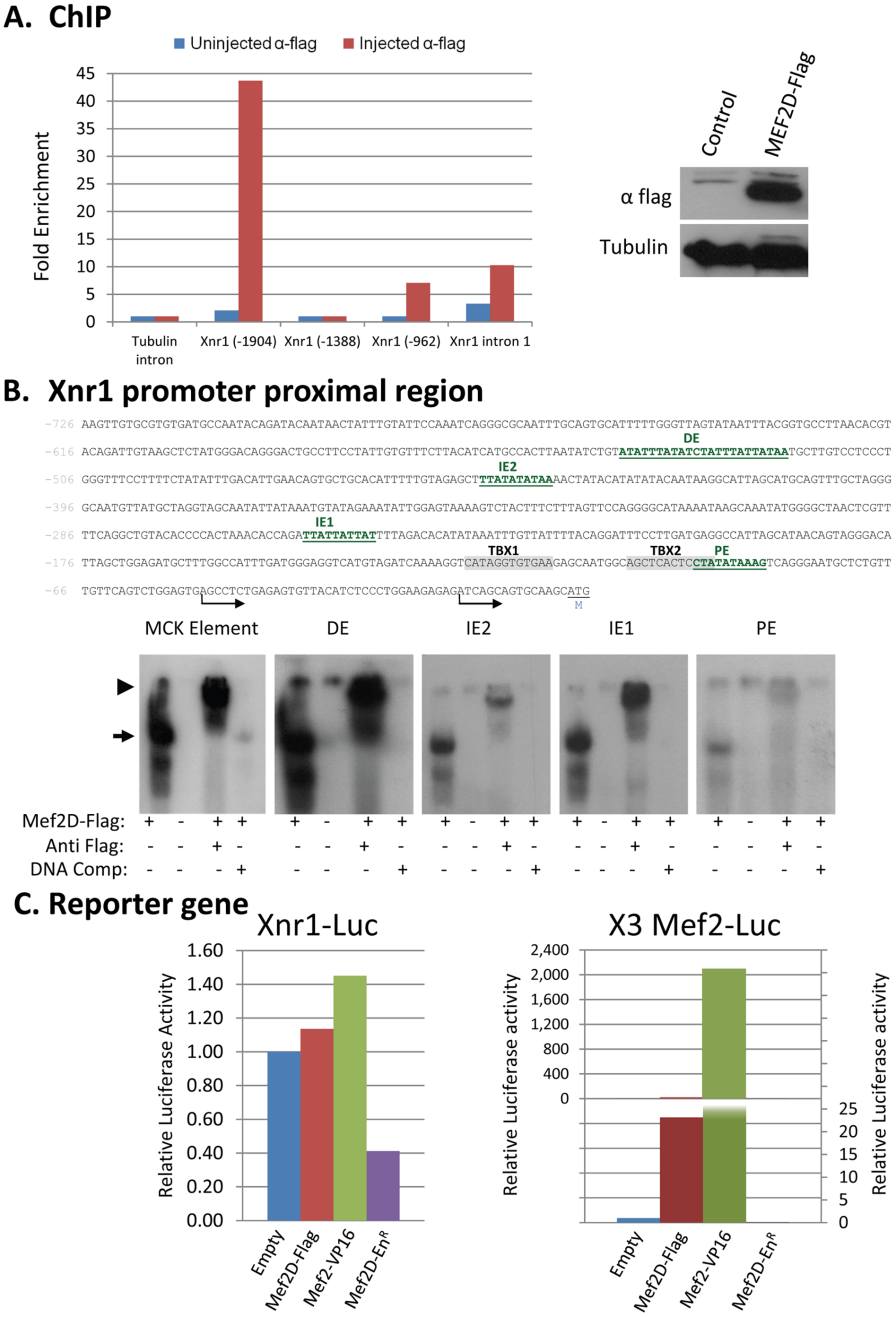
MEF2D associates with *Xnr1* regulatory elements. (A) Chromatin immunoprecipitation (ChIP): Embryos were injected with mRNA encoding MEF2D-Flag and at stage 10, crosslinked sheared chromatin was prepared. Chromatin was immunoprecipitated with anti-Flag (polyclonal, Sigma) or with pre immune serum (control) and was subjected to a qPCR reaction with several pairs of primers (left). Expression of the injected MEF2D-Flag protein was analyzed by Western blot (right). (B) Upper panel: *Xnr1* promoter sequence (proximal region) with highlighted putative binding sites of MEF2. PE-proximal element; IE1, 2-Intermediate element 1, 2; DE-distal element; TBX1, 2- T box binding sites (VegT) [47]. Arrows show the two transcription start site and “M” the translation initiation codon. Lower panel: EMSA of each of the MEF2 binding elements coupled with protein extracts of stage 9 control embryos as well as embryos injected with *mef2d-flag* mRNA. Anti-flag antibody (1 µl, 0.1 µg/µl) was included in some reaction mixtures while unlabeled homologous double stranded oligonucleotides in 100 fold excess over the probe was included in others, as indicated. Unbound probes are not shown. Arrow indicates the MEF2D-DNA complex. Arrowhead indicates the Anti MEF2-MEF2D-DNA complex. (C) 293T HEK cells were transfected as indicated. Thirty six hours later, proteins were extracted and luciferase activity was measured and was normalized to total protein levels. Activity of the reported gene with an empty vector was set to a value of 1 and values of other treatments were standardized accordingly. Means of two independent experiments are presented.

We next characterized the effects of gain of MEF2 activity. To induce ectopic MEF2 activity, a transcript encoding MEF2D-Flag protein was marginally-injected to vegetal blastomeres of 4 cell embryos. Injection of this transcript induced the expression of a MEF2-dependent reporter gene (x3 MEF2-Luc) (see figure 8C). Injected *mef2d-flag* mRNA caused ectopic punctate staining of *Xbra* in the vegetal region as observed in *in situ* hybridization analysis (Figure 4A, left panel). MEF2D-Flag slightly increased the expression of endogenous mesoderm and organizer genes as shown by RT-PCR analysis (Figure 4A, right panel). In a second approach to induce ectopic MEF2 activity, a transcript encoding the potent HSV VP16 activation domain and the DNA binding domain of MEF2C (*mef2-vp16*) was marginally injected to embryos (Figure 4B). The constitutively active MEF2-VP16 chimera protein induced prominent ectopic vegetal expression of *Xbra* in gastrula stage embryos. Injection of *mef2-vp16* mRNA into one of two blastomeres expanded the expression domain of *myod* in the injected half of the embryo at the neurula stage. Marginal zone injection of *mef2-vp16* mRNA induced the expression of *Xnr1* in gastrula VMZ explants and of *myod* and *muscle actin* in neurula VMZ explants. Overall, these results indicated that exogenously-expressed MEF2 protein induced ectopic expression of paraxial mesoderm genes.

### FGF8 Induces the Expression of MEF2D in the Marginal Zone

**Figure 5:**
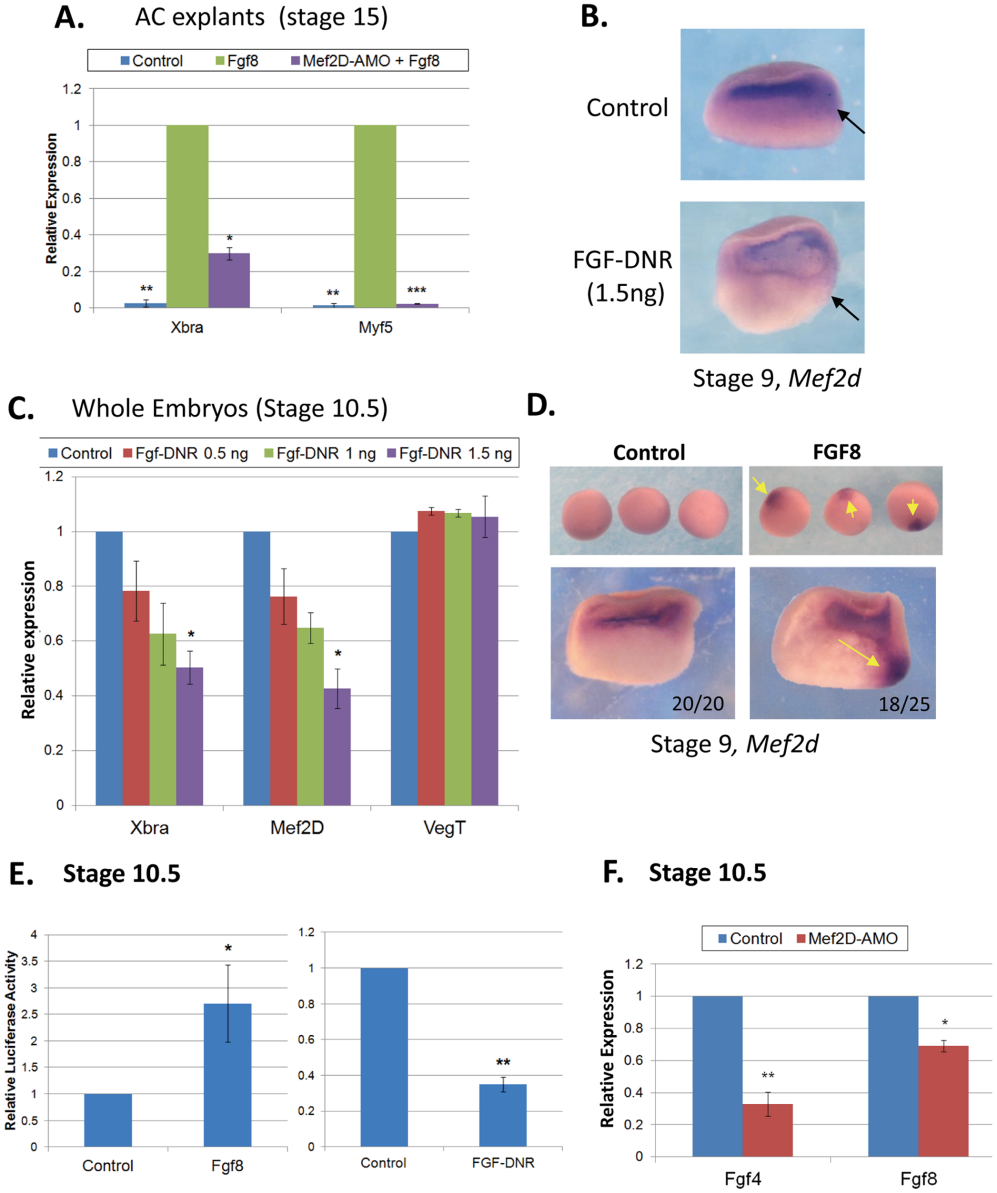
FGF signaling and MEF2D cooperate in mesoderm specification. (A) *fgf8* mRNA was injected without or with MEF2D AMO to one cell embryos. At stage 8, AC explants were isolated and grown to stage 15. RNA was extracted and gene expression was analyzed by qPCR reaction. Expression levels of each gene induced by FGF8 were arbitrarily set to a value of 1, and values of other treatments were standardized accordingly. Data are presented as means ± SE of two independent experiments with duplicates(B) mRNA encoding dominant negative FGF receptor (FGF-DNR) was marginally injected to one cell embryos and at stage 9 hemisected embryos were analyzed by ISH with a probe to *mef2d.* (C) mRNA encoding FGF-DNR was marginally injected to one cell embryo at the indicated concentrations and qPCR analysis was performed on isolated RNA from stage 10.5 embryos. Expression levels of each gene in uninjected embryos were arbitrarily set to a value of 1, and values of other treatments were standardized accordingly. Data are presented as means ± SE of two independent experiments with duplicates. (D) *fgf8* mRNA was injected vegetally to one of four cell embryos. At stage 9 embryos were analyzed by ISH with a probe to *mef2d.* Upper panel: whole embryos. Lower panel: hemisected embryos. (E) One cell embryos were injected with a plasmid containing x3 Mef-Luc reporter gene without or with *fgf8* mRNA or FGF-DNR mRNA. At stage 10.5 proteins were extracted and luciferase activity was measured and normalized according to the total amount of proteins. Data are presented as means ± SE of three independent experiments with duplicates. (F) qPCR analysis of control embryos and embryos injected with MEF2D AMO. Five embryos on each group were injected and RNA was extracted at stage 10.5. Expression levels of control embryos were arbitrarily set to a value of 1, and values of injected embryos were standardized accordingly. Data are presented as means ± SE of two independent experiments with duplicates.

Since MEF2D-Flag activates only minimally the expression of mesodermal genes relative to MEF2-VP16, we hypothesized that MEF2 may cooperate with one of the signaling events that induces mesoderm. It was demonstrated before that marginal FGF signaling at the late blastula stage initiates mesodermal gene expression [10], [11], [12], [13], [14], [15]. Therefore, we investigated how FGF signaling and MEF2D interact to elicit mesoderm gene expression. First, we inquired whether MEF2D protein was required for FGF8-induced mesoderm gene expression (Figure 5A). As expected, injection *of fgf8* mRNA induced the expression of mesodermal genes, *Xbra* and *myf5* in AC explants. Co- injection of *fgf8* mRNA and MEF2D AMO significantly reduced the expression of mesodermal genes, indicating that MEF2D is necessary for the induction by FGF8 of mesoderm gene expression. Next, we investigated whether FGF signaling affected *mef2d* gene expression levels (Figure 5B). Dominant negative FGF receptor (XFD) was ectopically expressed in embryos and the expression of *mef2d* was followed by ISH (Figure 5B) and qPCR (Figure 5C). The marginal expression of *mef2d* was reduced in *xfd*-injected embryos while the animal expression appeared to remain unaffected in stage 9 embryos (Figure 5B). qPCR analysis of stage 10.5 embryos revealed that injected *xfd* mRNA significantly reduced the expression of *mef2d* as well as the expression of *Xbra* without affecting the expression of *vegt* (Figure 5C). In a gain-of-function experiment, injection *of* fgf8 mRNA into vegetal blastomeres induced robust ectopic and localized expression of *mef2d* (Figure 5D). Next, we inquired whether the activity of MEF2D was affected by FGF signaling. MEF2 activity was monitored by measuring luciferase activity of injected x3 MEF2-Luc reporter gene. Co-injection *of xfd* mRNA together with the reporter gene reduced whereas *of fgf8* mRNA increased the reporter activity (Figure 5E). We conclude therefore that FGF signaling sufficed to induce *mef2* expression and activity. Further insight into the effect of MEF2D on FGF expression was gained by observing that injected MEF2D AMO significantly *reduced fgf4* and *fgf8* expression at late blastula (Figure 5F). Overall, our results indicated the existence of a positive feedback loop between FGF and MEF2D in the marginal zone that participates in the early expression of mesoderm genes.

### MEF2 Cooperates with Vegetal Signals to Induce Mesoderm Gene Transcription

**Figure 6:**
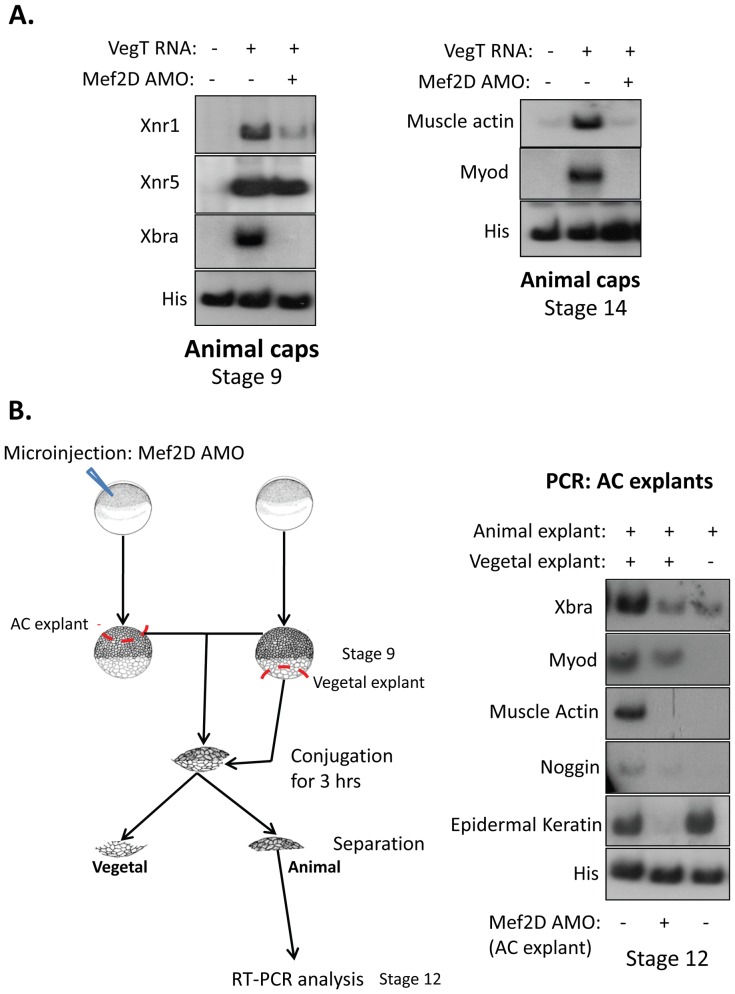
Animal cap- MEF2D-depleted explants do not express mesoderm markers in Nieuwkoop recombinants. (A) Embryos were injected with *vegt* mRNA without or with MEF2D AMO. AC explants (10 explants per treatment) were dissected at stage 8 and were allowed to grow to stage 9 (left) or to stage 14 (right). RNA was extracted and semi quantitative RT-PCR was performed. (B) Left panel: Scheme of the experiment. Animal cap explants from control or AMO-injected embryos were dissected at stage 9 and combined with stage 9 vegetal explants for 3 hours (n = 20). Following a co-culture period, explants were separated and AC explants grown to stage 12. RNA was extracted and analyzed by semi quantitative RT-PCR. Right panel: Expression of mesoderm genes was induced in control AC explants previously combined with vegetal explants (left lane) but was barely induced in AC explants from AMO-injected embryos (middle lane).

Next, we asked whether MEF2D functions as a mediator of vegetal signals in the initiation of mesoderm gene expression. Injection of *vegt* mRNA induced the expression of pro-mesodermal genes in control AC explants [28], yet expression of these genes by *vegt* mRNA was prevented in MEF2D-knockdown embryos (Figure 6A). Injected MEF2D AMO prevented the expression of *Xnr1* and *Xbra* at a late blastula stage and *myf5* and *muscle actin* at a late gastrula stage. We therefore conclude that MEF2D is necessary for VegT-induced expression of mesoderm genes.

Since MEF2 is expressed in the marginal zone at the late blastula stage, it may function *in situ* to induce the expression of mesodermal genes. To test this possibility, we used a recombinant explant experiment [29] in which vegetal explants from control embryos were combined with animal cap (AC) explants from control or MEF2D AMO-injected late blastula stage embryos. After a 3 hour co-culture period, animal caps were separated from vegetal masses, cultured until sibling embryos reached late gastrula stage and analyzed for the expression of mesodermal genes (Figure 6B, left panel). As expected, the expression of mesodermal genes *Xbra*, *myod, muscle actin* and *noggin* was induced in AC explants from wild type embryos that were co-cultured with vegetal explants (Figure 6B, right panel). In addition to mesodermal genes, the animal-specific gene, *Epidermal keratin*, was expressed in AC explants. However, the same mesodermal genes were only mildly induced in AC explants that originated from MEF2D-depleted embryos. Interestingly, the expression of *Epidermal keratin* was diminished in MEF2D-depleted AC explants. This result indicated that *in situ* expression of MEF2D protein in the animal cells was necessary for their response to the vegetal mesoderm-inducing signals and to the expression of animal ectoderm marker, *Epidermal keratin*.

### MEF2D Binds Directly to Xnr1 Transcription Regulatory Sequences

**Figure 7:**
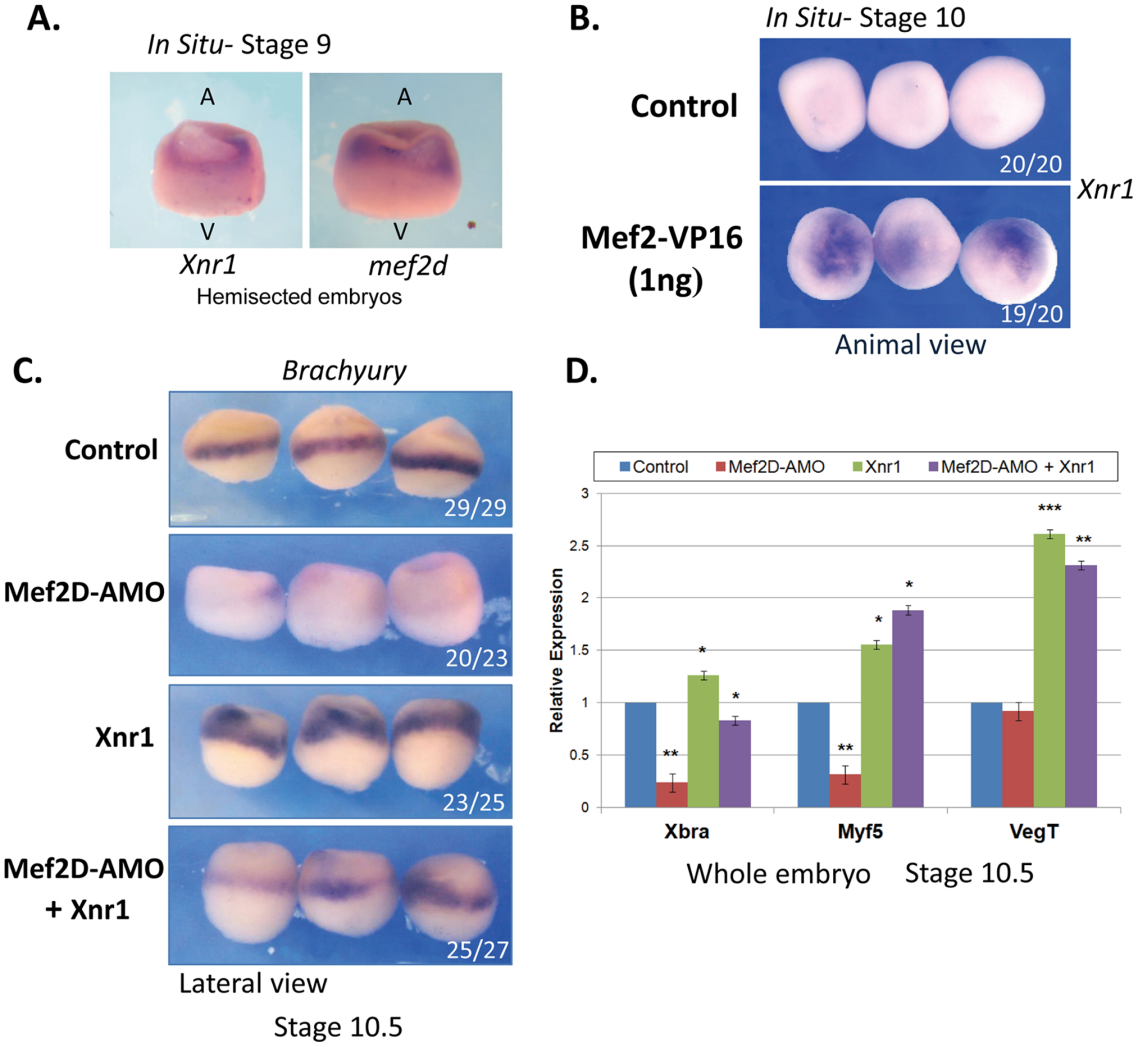
MEF2 activity is sufficient to induce the expression of *Xnr1* that partly mediates the function of MEF2D in mesoderm gene expression. (A) ISH analysis of hemisected embryos at stage 9 using probes to *xnr1* (left) and *mef2d* (right). (B) *Mef2-vp16* encoding mRNA was injected marginally to one cell embryos. Stage 10 embryos were analyzed by ISH using an *Xnr1* probe. The animal side is presented. (C) One cell embryos were injected with mRNA encoding *Xnr1*, MEF2D-AMO or both. Stage 10.5 embryos were analyzed by ISH using antisense *Brachyury* probe. (D) Injections were performed as in C (each treatment; 18 embryos). RNA was extracted from stage 10.5 embryos and qPCR was performed. Data are presented as means ± SE of three independent experiments with duplicates.

One of the targets of MEF2D that were identified in this work was the *Xnr1* gene whose expression was initiated at MBT (stage 8). We could expect therefore similar expression patterns *of mef2d* and *Xnr1*. Indeed, ISH analysis indicated that at late blastula these two genes are expressed marginally and to a lesser extent vegetally (Figure 7A) *Xenopus* Nodal-Related (*Xnr*) genes are required for mesendoderm induction and gastrulation movements [30]. Next, we asked whether MEF2 activity was sufficient to induce ectopic *Xnr1* expression. Marginal injection of *mef2-vp16* mRNA induced robust ectopic animal expression of *Xnr1* (Figure 7B) and low *Xnr1* expression in the vegetal region (data not shown). Thus, MEF2 activity was sufficient to induce *Xnr1* expression in the animal but not in the vegetal region. We next examined whether Xnr1 was able to substitute MEF2D activity in the induction of mesoderm genes. Injection of *Xnr1* mRNA induced significant expression of *Xbra* both marginally and animally while injection of MEF2D-AMO completely abolished physiological marginal *Xbra* expression (Figure 7C). Injection of both MEF2D-AMO and *Xnr1* mRNA induced intermediate levels of *Xbra* expression. Similarly, injected *Xnr1* rescued the expression of *Xbra* and *Myf5* in MEF2D-depleted embryos as was analyzed by qPCR (Figure 7D). Moreover, injection of *Xnr1* mRNA also induced ectopic mesodermal gene expression in AC explants even in the presence of co-injected MEF2D AMO (Figure S4). These results indicated that *Xnr1*, one of MEF2D target genes, mediated at least some of MEF2D activities in mesoderm specification. Next, we asked whether MEF2D associates directly with *Xnr1* regulatory sequences at the chromatin level. To explore this question we employed chromatin immunoprecipitation (ChIP) assay in embryos that were injected with mRNA encoding for MEF2D-Flag protein (Figure 8A). Fragmented chromatin was immunoprecipitated (IPed) with either anti-Flag (specific) or pre immune (nonspecific) serum and qPCR analysis of fragments scattered along the *Xnr1* gene was performed. IP with anti-Flag antibodies resulted in a significant and reproducible enrichment of fragments found around 1900 (distal) and 900 (proximal) base pairs upstream to *Xnr1* translation initiation site and within the first intron of *Xnr1* as compared to IP with pre-immune serum (Figure 8A). Intron sequences of the *tubulin* gene and other upstream regions of the *Xnr1* gene were not enriched in anti-Flag complexes relative to pre-immune complexes indicating the specificity of the assay. We conclude, therefore, that MEF2D associates with several regulatory regions of the *Xnr1* gene. Sequence analysis revealed several putative Mef2 binding sites in the different regions that were enriched in the ChIP analysis. Using electrophoretic mobility shift assay (EMSA), we further analyzed the *in vitro* MEF2 binding to 4 elements located within 600bp from the translation initiation site (proximal region) (Figure 8B) [31], [32]. For that purpose, protein extracts were prepared from embryos injected with mRNA encoding the MEF2D-Flag pr*o*tein and incubated with four different DNA probes, each containing one of the identified elements. In all four cases, a shifted band representing a Mef2-DNA complex was detected. The most proximal element (PE) displayed the lowest binding whereas the most distal element (DE) exhibited the highest binding efficiency. Binding of MEF2D-Flag to any of these elements appeared weaker than its binding to the optimal element of the MCK enhancer (Figure 8B). The *in vitro* binding to the proximal region (between −540 and −230 relative to translation initiation site) possibly corresponds to the enrichment of the −900 fragment in the ChIP assay, in which the average sonicated fragment size was around 500–1000 bps (data not shown). A reporter gene carrying these elements (696bp upstream regulatory region) was not activated by MEF2D-Flag both in embryos (data not shown) and in transfected cells (Figure 8C). The same reporter gene was only mildly activated by exogenous MEF2-VP16 and was significantly repressed by the Mef2 repressor protein, MEF2D-En^R^. In contrast, the activity of a control reporter containing three optimal Mef2 binding sites (x3 Mef2-Luc) was potently activated by MEF2D-Flag or by MEF2-VP16 and was repressed by MEF2D-En^R^. We conclude, therefore, that although the proximal promoter contains Mef2 binding sites, this region is not sufficient for transcriptional activation of the *Xnr1* gene by MEF2D.

## Discussion

### Dynamic Expression of MEF2 Proteins at Early Developmental Stages

The present study aimed at understanding expression patterns and roles of MEF2 proteins during blastula to gastrula stages. We find that MEF2 expression pattern is very dynamic: before MBT, maternal MEF2A and MEF2D proteins are localized in the animal hemisphere. Following MBT, the expression of zygotic MEF2D expands primarily to the marginal region whereas MEF2A expression declines and is initiated at later neurula stages. Our study focused, therefore, on the role of post-MBT MEF2D expression in the marginal region. As is expected from the dynamic early expression of MEF2D, the phenotype of MEF2D-depleted embryos is complex; gastrulation is delayed, embryos do not properly elongate and axial structures of mesoderm and ectoderm origins are absent. The present investigation focused on the mode of MEF2D involvement in mesoderm gene expression, leaving for future studies the issue of ectoderm gene expression. Results presented in this study indicate that MEF2D plays a major role in mesoderm specification.

### MEF2D Affects Mesoderm Specification and Patterning

Mesoderm induction occurs after MBT when zygotic transcription begins under the influence of vegetally-localized maternal molecules [33], [34]. The immediate marginal expression of MEF2D following MBT suggested its involvement in mesoderm specification. Knockdown of MEF2D reduced the expression of a wide array of mesodermal genes encoding for signaling molecules and transcription factors that affect early mesoderm. The expression of marginal zygotic genes such as *fgf8, Xbra, vent1, Xnr1* and *goosecoid* diminished whereas the expression levels of vegetal genes; *Xnr5, mixer* and *vegt* was not affected. These results indicate that MEF2D is an early zygotic pan-mesodermal master gene which is involved directly or indirectly in the expression of majority of marginal genes. A possible explanation for this wide effect of MEF2D is a likely reflection of its regulatory role in the expression of critical signaling molecules of the FGF, Nodal, BMP and Wnt signaling pathways that are essential for mesoderm specification and patterning.

The maternal VegT transcription factor is directly involved in the vegetal expression of Nodal ligands that induce mesoderm in the overlaying marginal cells [7], [33], [34]. We show that MEF2D expression is required in an animal explant for its response to vegetal signals in the initiation of mesoderm gene transcription. MEF2D is, therefore, a marginally-localized transcription factor that transforms vegetal signals to mesoderm gene expression. MEF2D may cooperate with vegetal signals of Xnr5 and Xnr6 in a mechanism involving interaction with the downstream Nodal effector, Smad2 at the regulatory regions of their target genes [7], [35]. Alternatively, MEF2D could be phosphorylated by a kinase of non-canonical Nodal signaling [19], [36]. Thus, MEF2D may be viewed as a hub that integrates vegetal-originated signals into marginal transcription program of the mesoderm.

### MEF2D and FGF Function in a Positive Feedback Loop to Induce Mesoderm Specification

FGF signaling plays a crucial role in mesoderm specification [10], [11], [12], [13], [14], [15]. Similarly to MEF2D, several FGF ligands such as FGF2, FGF4 and FGF8 are expressed in the marginal zone of late blastula stage embryos and in the DMZ of early gastrula stage [37]. Little is known about transcription factors that act downstream of FGF signaling in *Xenopus* during mesoderm formation. One downstream effector of FGF is the SRF-interacting Ets family member Elk-1, which is phosphorylated in response to FGF signaling and is required for *Brachyury* expression [38]. Our results indicate that MEF2D is a newly identified downstream target of FGF signaling. We show that FGF signaling is required for the expression of MEF2D in the marginal zone. Loss of FGF activity prevents marginal expression of MEF2D, whereas exogenous FGF activity induces ectopic MEF2D expression in the vegetal region. Moreover, in the absence of MEF2D expression, FGF failed to induce mesodermal gene expression in animal cap assay. Presently, we do not know if MEF2D is phosphorylated in response to FGF signaling in *Xenopus*. This possibility is likely since in *Xenopus*, FGF signals through MAPK to induce mesoderm [39], [40], [41] and MEF2 proteins are known to be phosphorylated by mitogen activated protein kinase (MAPK) pathways [42], [43], [44], [45], [46]. Notably, it is not just that FGF affects MEF2D expression but also reciprocally MEF2D is required for the expression of FGF ligands. Therefore, FGF and MEF2D maintain each other overlapping expression in the forming mesoderm at blastula to gastrula stages.

### MEF2D Associates with Regulatory Sequences of xnr1 Gene

Regulation of *Xnr1* transcription was thoroughly investigated. *Xnr1* is expressed from blastula stages in the vegetal marginal border, and at the gastrulation stage it is predominantly expressed in the dorsal marginal zone. *Xnr1* is regulated by maternal factors VegT and β catenin, and zygotic Nodal signaling (Xnr5, and 6). *Xnr1* upstream regulatory sequences were demonstrated to be directly regulated by VegT and effectors of Wnt signaling (TCF/LEF) [47]. The first intron contains activin response element (ARE) that was demonstrated to bind a complex of FoxH1 and Smad2 [35]. Our results indicate that MEF2D may be added to the growing list of transcription factors regulating *Xnr1* expression. Evidence supports the notion that *Xnr1* is a target gene of MEF2D. First, knockdown of MEF2D diminishes *Xnr1* expression while excessive Mef2 activity induces ectopic *Xnr1* expression in the animal region. Second, ectopic expression of Xnr1 partly rescues mesoderm gene expression in the absence of MEF2D. Third, chromatin IP analysis indicates that MEF2D associates with several regions along the *Xnr1* gene, both upstream to the transcription start sites and within its first intron. These findings indicate a complex regulation of *Xnr1* transcription by MEF2D since several regions along the gene associate with MEF2D, and probably the combinatorial activity of distinct regions is necessary to activate transcription. In the present study we focused on the proximal promoter region that contains several putative MEF2 binding sites (YTA(A/T)_4_TAR). Our results demonstrate the *in vitro* binding of MEF2D to some of these sites. Some of the putative MEF2 sites are adjacent to previously identified sites of VegT and FoxH1/Smad2, indicating a possible cooperation between the different transcription factors in the regulation of *Xnr1* transcription. Transcription from the same proximal promoter region was not induced by expression of MEF2D, but was repressed by the MEF2 repressor protein, MEF2D-En^R^. Therefore, we conclude that MEF2 binds to sites at the proximal promoter region and is necessary but not sufficient to drive transcription from this element. Future studies will clarify whether binding of the MEF2D protein to additional regions of the *Xnr1* gene that were identified by the ChIP assay play a role in *Xnr1* transcription.

### Complex Activities of MEF2 Proteins during Early Stages of Development

**Figure 9:**
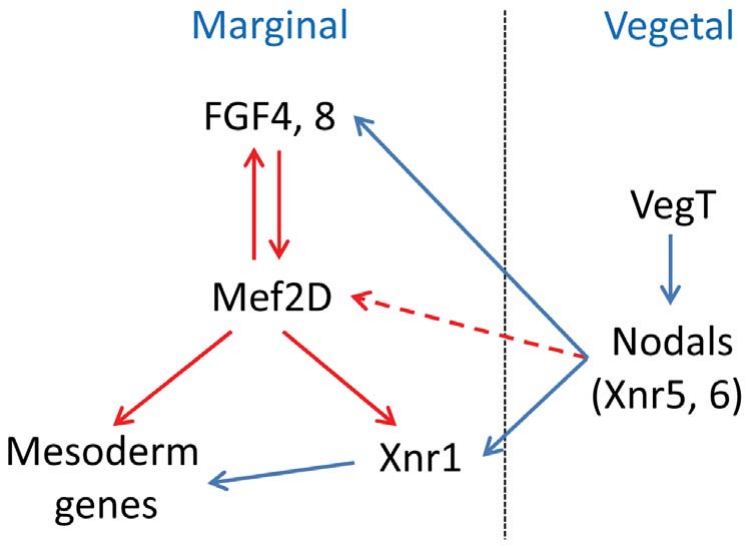
A model describing the role of MEF2D in mesoderm gene expression. Solid arrows indicate activation at the levels of expression and/or activity. Dashed arrow indicates the cooperation between Nodal signaling and MEF2D activity in marginal mesoderm activation. Red arrows indicate conclusions of the present study while blue arrows indicate previous knowledge.

While previous studies concentrated on late developmental activities of MEF2 proteins, in this study we analyzed expression and function of MEF2 proteins from fertilization to early gastrula stages. Expression patterns of MEF2 proteins indicate their participation in ectoderm and mesoderm specification. This study of the role of MEF2D in early mesoderm introduces new components to the already complex network of molecules that participate in mesoderm formation (Figure 9). We suggest that following MBT MEF2D is involved in the intricate array of signaling pathways affecting mesoderm. FGF signaling regulates marginal expression of MEF2D which is necessary to mediate FGF activity in mesoderm gene expression. Also, MEF2D is required for the expression of FGF ligands (FGF4, 8). Therefore, FGF and MEF2D function in a positive autoregulatory loop to maintain marginal mesoderm gene expression. The marginal expression of MEF2D is necessary to mediate mesoderm gene expression by vegetal Nodal signaling. MEF2D induces the transcription of *Xnr1* and some other mesodermal genes, and the Xnr1 protein partly mediates the mesodermal regulatory function of MEF2D. Overall, MEF2D responds to and activates signals necessary for mesoderm specific transcription. Global analysis of MEF2 target genes is expected to reveal a much broader role for this family of transcription factors as was demonstrated in *Drosophila* [48]. Several interesting questions that arise from this work remain to be explored; which additional developmental programs are affected by MEF2 at these early stages and how? Which genes are direct targets of MEF2 proteins? Is the early role of MEF2 in germ layer specification conserved between species? Our ongoing investigations focus on some of these questions.

## Supporting Information

Figure S1 **Expression of MEF2D in paraxial mesoderm of stage 14 embryos.** Transverse section (left) and longitudinal section (right) of stage 14 embryos were reacted with anti-MEF2 antibodies (orange) and counterstained with hematoxylin. Nuclear staining of MEF2 is primarily observed in paraxial mesoderm.
(TIF)

Figure S2 **Expression patterns of** ***mef2a*** **and** ***mef2d.*** ISH was performed on embryos at different gastrula to neurula stages with probes to *mef2a* and *mef2d.*
(TIF)

Figure S3 **Dominant negative MEF2D protein reduces mesodermal gene expression.** mRNA encoding MEF2D-engrailed (MEF2D-En^R^) chimera was co-injected to one cell embryos with a x3 MEF2-Luc reporter gene. Luciferase activity was measured in extracts of stage 10.5 embryos (n = 18) (left panel). Increasing amounts of MEF2D-En^R^ mRNA were injected to one cell embryos as indicated, and RNA levels of several genes were analyzed at stage 10.25 by semi-quantitative RT-PCR (right panel).
(TIF)

Figure S4 **Xnr1 can induce mesoderm gene expression in MEF2D-depleted animal cells.** *Xnr1* mRNA was injected to one cell embryos without or with MEF2D AMO. AC explants were dissected at stage 8 and were grown to stage 10.5. RNA was extracted and qPCR was performed. Data are presented as means ± SE of three independent experiments with duplicates.
(TIF)
