# Supplementary figures and images for: Transcriptional Regulation of Mesoderm Genes by MEF2D during Early Xenopus Development
Source: PLoS One. 2013 Jul 19;8(7):e69693. doi: 10.1371/journal.pone.0069693 (PMC3716644; doi:10.1371/journal.pone.0069693)

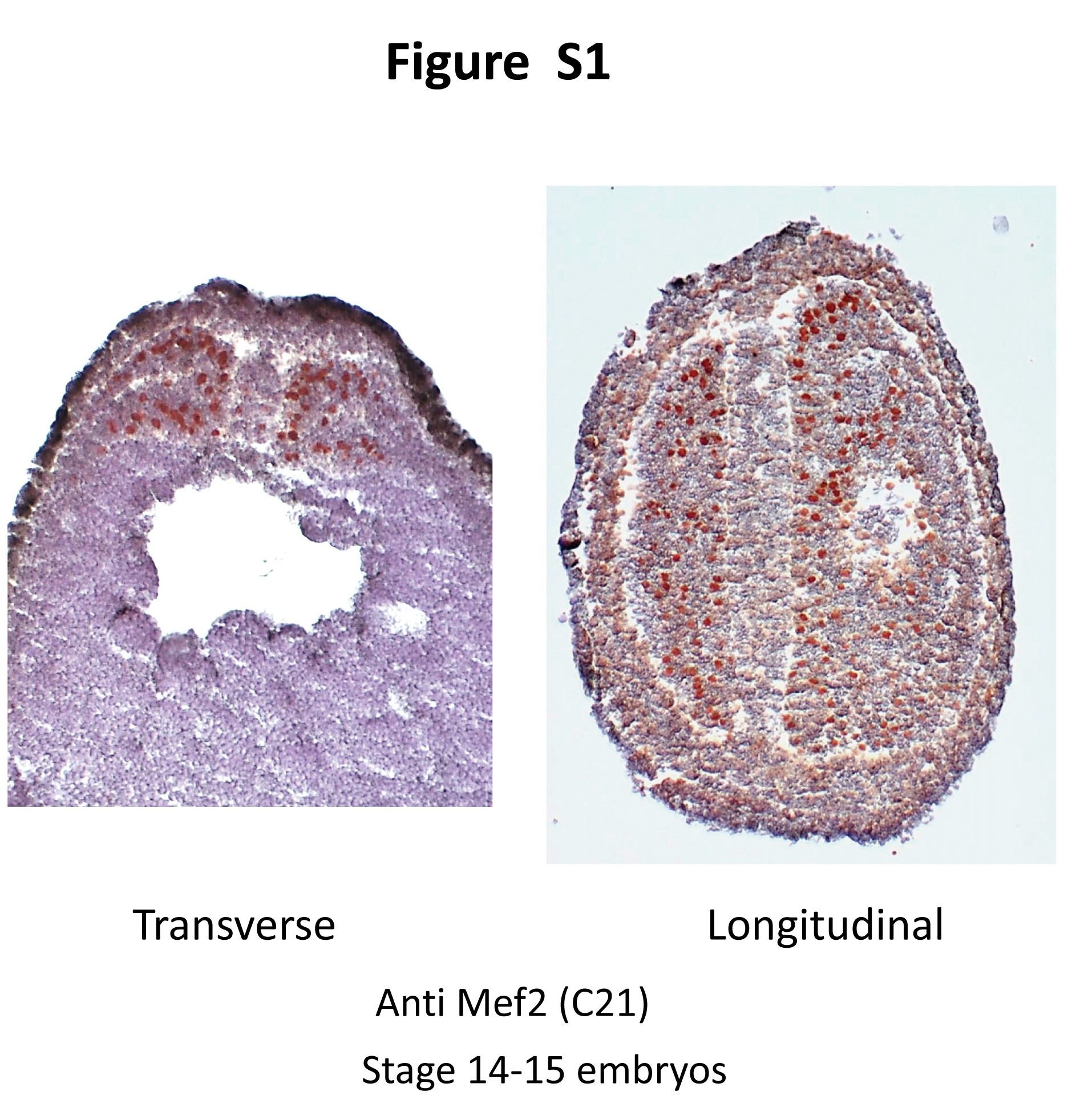

Supplement: Figure S1 — Expression of MEF2D in paraxial mesoderm of stage 14 embryos. Transverse section (left) and longitudinal section (right) of stage 14 embryos were reacted with anti-MEF2 antibodies (orange) and counterstained with hematoxylin. Nuclear staining of MEF2 is primarily observed in paraxial mesoderm. (TIF) [file pone.0069693.s001.tif]

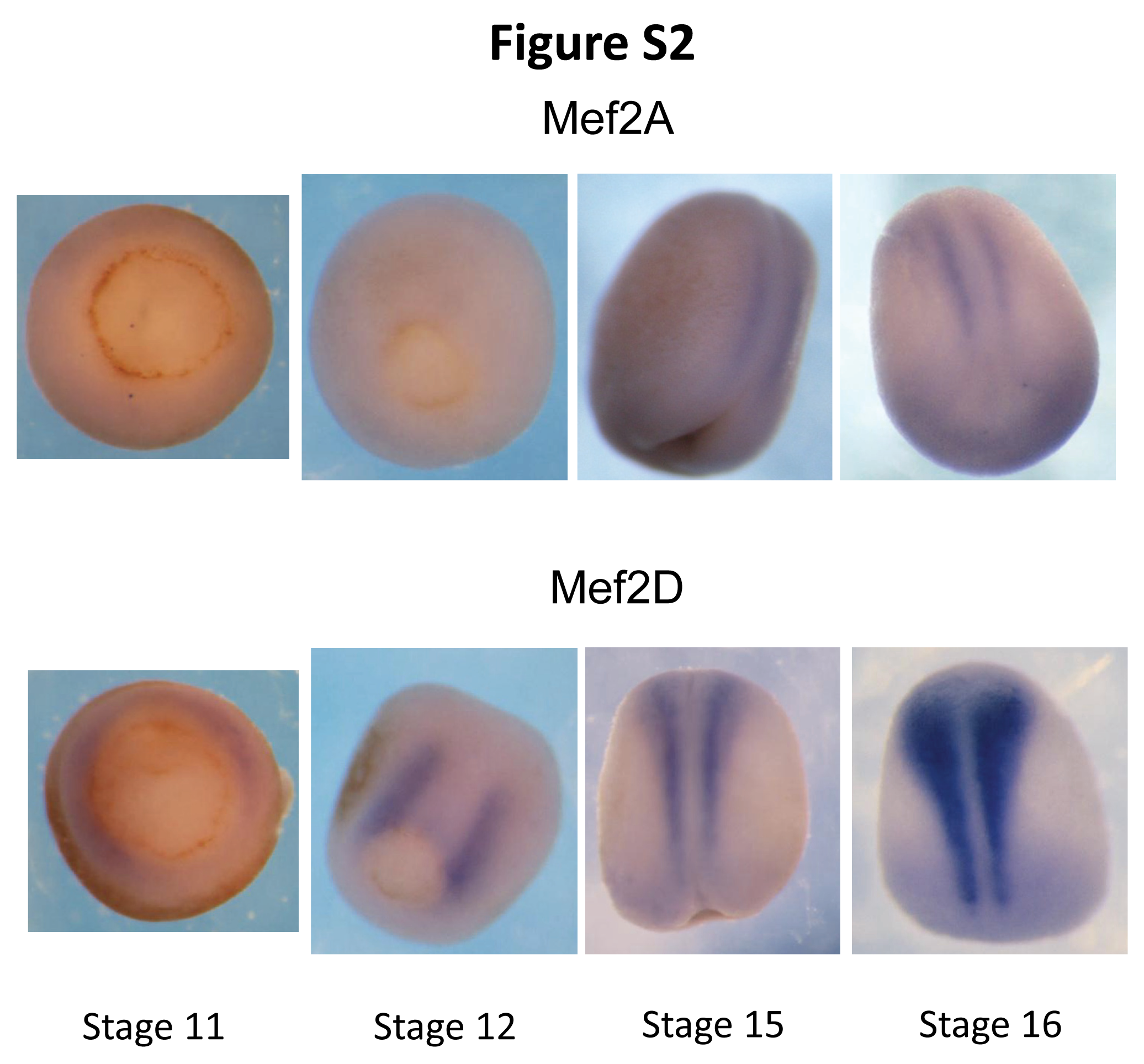

Supplement: Figure S2 — Expression patterns of mef2a and mef2d. ISH was performed on embryos at different gastrula to neurula stages with probes to mef2a and mef2d. (TIF) [file pone.0069693.s002.tif]

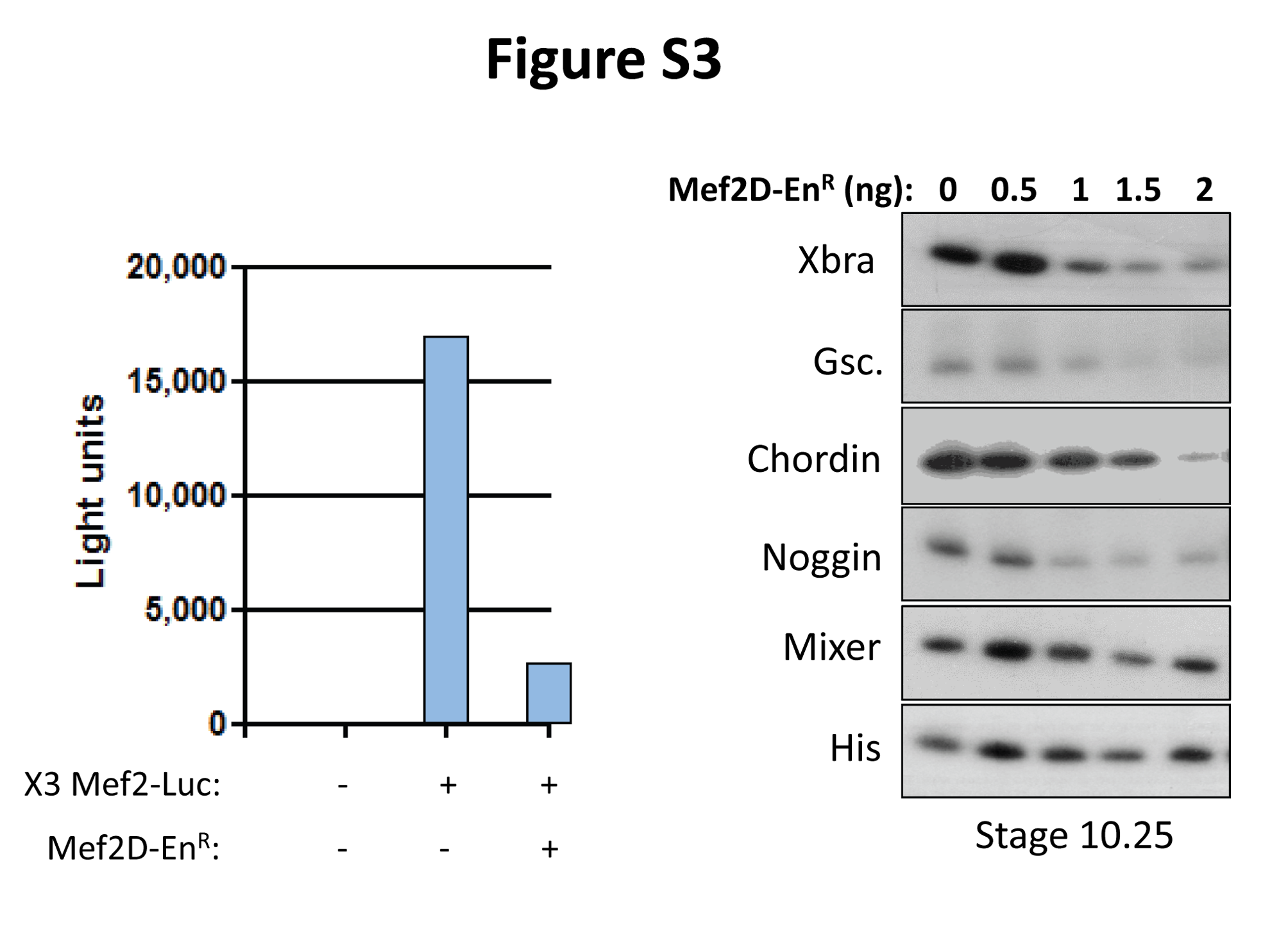

Supplement: Figure S3 — Dominant negative MEF2D protein reduces mesodermal gene expression. mRNA encoding MEF2D-engrailed (MEF2D-EnR) chimera was co-injected to one cell embryos with a x3 MEF2-Luc reporter gene. Luciferase activity was measured in extracts of stage 10.5 embryos (n = 18) (left panel). Increasing amounts of MEF2D-EnR mRNA were injected to one cell embryos as indicated, and RNA levels of several genes were analyzed at stage 10.25 by semi-quantitative RT-PCR (right panel). (TIF) [file pone.0069693.s003.tif]

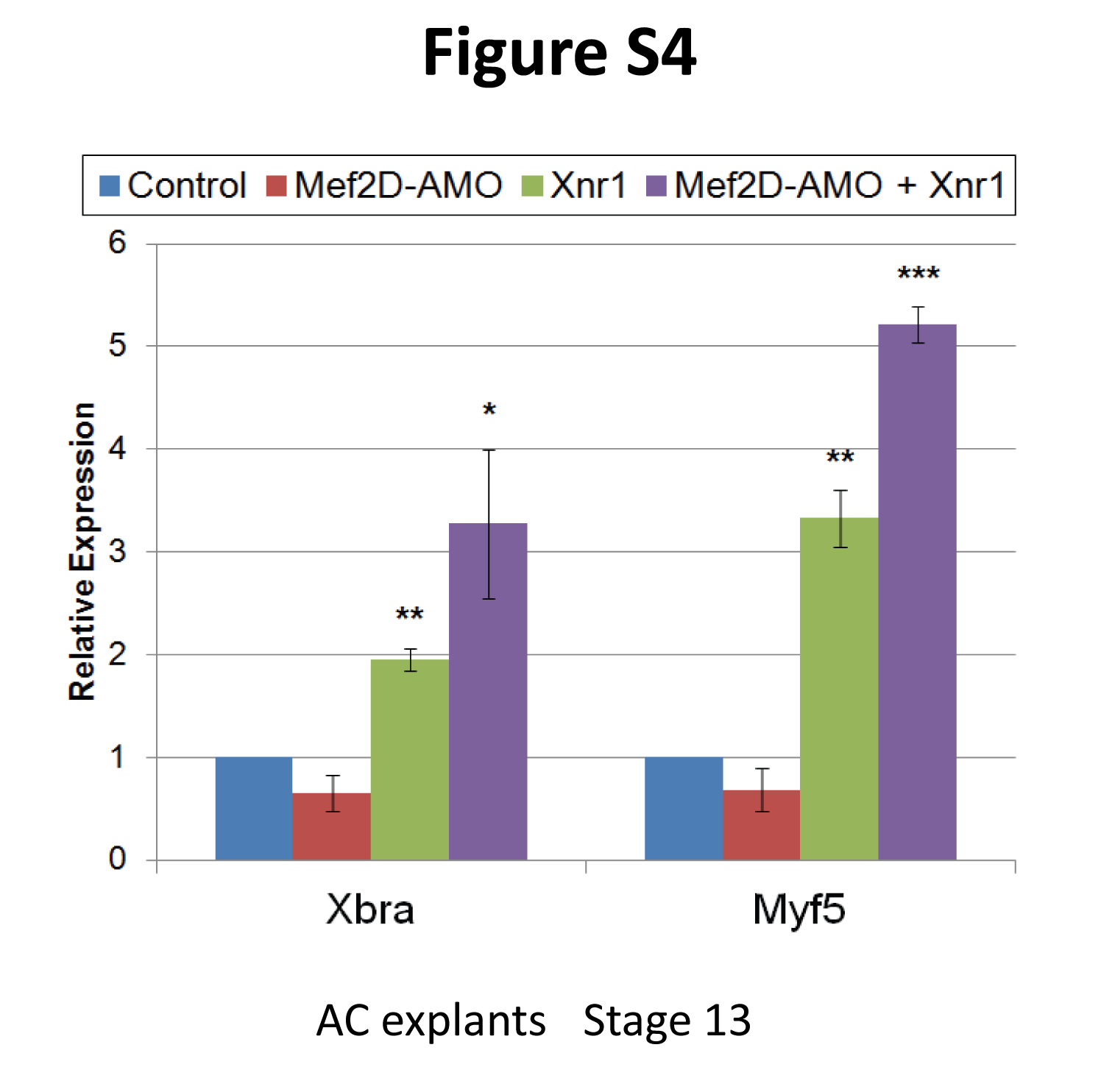

Supplement: Figure S4 — Xnr1 can induce mesoderm gene expression in MEF2D-depleted animal cells. Xnr1 mRNA was injected to one cell embryos without or with MEF2D AMO. AC explants were dissected at stage 8 and were grown to stage 10.5. RNA was extracted and qPCR was performed. Data are presented as means ± SE of three independent experiments with duplicates. (TIF) [file pone.0069693.s004.tif]
